# Elevated pretreatment platelet distribution width and platelet count predict poor prognosis in nasopharyngeal carcinoma

**DOI:** 10.18632/oncotarget.22528

**Published:** 2017-11-20

**Authors:** XueCheng Xie, XiaoChun Zeng, SuJuan Cao, XiaoMao Hu, QiaoJing Shi, Dan Li, ShiYuan Zhou, Ping Gu, ZhongShan Zhang

**Affiliations:** ^1^ Department of Oncology, The Affiliated Hospital of XiangNan University, Chenzhou, China; ^2^ Department of Nuclear Medicine, The Affiliated Hospital of XiangNan University, Chenzhou, China; ^3^ Department of Child Healthcare, Chenzhou First People's Hospital, Chenzhou, China

**Keywords:** nasopharyngeal carcinoma, platelet distribution width, prognosis, overall survival

## Abstract

**Background:**

Previous studies have demonstrated that platelets play a multifaceted role in cancer progression and metastasis. However, the value of platelet indices for predicting survival in nasopharyngeal carcinoma (NPC) patients remains unknown. The aim of this study was to evaluate the predictive significance of platelet indices in NPC cases.

**Materials and Methods:**

A total of 168 patients who were diagnosed with NPC between January 2011 and June 2012 were recruited. The optimal cut-off values for the platelet indices were determined using a receiver operating characteristic curve. The Kaplan-Meier method and Cox regression were used to evaluate the prognostic impact of the potential predictors.

**Results:**

Of the 168 patients, high platelet distribution width (PDW) and platelet count (PLT) levels were observed in 81 (48.21%) and 68 (40.48%) of the patients, respectively. An increased PDW was associated with the depth of invasion (T stage, P = 0.019), lymph node metastasis (N stage, P = 0.026), and clinical stage (P < 0.001). Moreover, the survival analysis showed that the overall survival of the patients with a PDW > 16.3 fL or platelet count > 266 × 10^9^/L was associated with a poorer prognosis (both P < 0.001). In the multivariate Cox regression model, the PDW (P < 0.001), PLT (P = 0.001), T stage (P < 0.001), N stage (P = 0.006), clinical stage (P = 0.005), and Epstein-Barr virus DNA (P = 0.039) were independent prognostic factors for the overall survival.

**Conclusions:**

The PDW and PLT are easily available via a routine blood test, and our study showed that the PDW and PLT could be prognostic predictors in NPC patients. However, further studies are required to confirm this conclusion.

## INTRODUCTION

Nasopharyngeal carcinoma (NPC) is the most frequently diagnosed tumor originating in the nasopharynx. It exhibits a distinct endemic distribution, with a particularly high incidence in Southern China and its surrounding regions [1]. Radiotherapy, with or without chemotherapy, is the standard treatment for patients with NPCs, while the prognosis for patients with tumor-node-metastasis (TNM) stages I and II is infusive [2]. Currently, TNM staging remains the gold standard for predicting the outcome of NPC [3]. However, even within the same staging category, there is variability in the patient outcomes because of the inability of the TNM system to reflect biological heterogeneity among the cancer cases [4]. Recent studies have reported a number of additional NPC prognostic markers, such as circulating Epstein-Barr virus (EBV) DNA loads [5], pretreatment serum lactate dehydrogenase [6], and microRNA signatures [7]. Due to the current limitations, such as detection difficulty, cost efficiency, and interlaboratory variability, the identification of appropriate and effective NPC prognostic markers is still of great value.

Multiple studies have suggested that platelets play a significant role in cancer progression and metastasis. For example, activated platelets promote cancer cell growth, aberrant angiogenesis, and invasion [8, 9]. Some platelet indices, such as the platelet count (PLT), mean platelet volume (MPV), platelet distribution width (PDW), and platelet-lymphocyte ratio (PLR), can be easily tested and have been demonstrated to be related to the prognosis of various cancers, including non-small cell lung cancer, breast cancer, gastric cancer, colorectal cancer, pancreatic cancer, and laryngeal cancer [10–15].

The MPV shows the average size of the platelets in the bloodstream, but it does not reflect the microscopically observed changes in the platelet size. The PDW is calculated as the coefficient of variation in the MPV, and high PDW values show that the change in the MPV is greater than normal [16]. Recently, some studies have found that an elevated pretreatment PLT or PLR was significantly and independently associated with a poor overall survival (OS) in NPC patients [17, 18].

These abovementioned studies only evaluated one or two biomarkers, without considering others. Moreover, the prognostic values of the PDW and MPV in NPC have not yet been established. Overall, the clinical significance of the platelet indices in the prognosis of NPC still remains unclear. Thus, the aim of this analysis was to investigate the prognostic value of the platelet indices mentioned above for patients with NPC, and to evaluate the correlation between these potential prognostic predictors and the clinical-pathological parameters.

## RESULTS

The patient characteristics are outlined in Table 1. Overall, there were 119 (70.83%) male patients and 49 (29.17%) female patients, and the median age at diagnosis was 49 years old (range 29–76). In terms of the staging system, 28 cases (16.67%) were categorized as stage I or stage II, and 140 patients (83.33%) were stage III or stage IV (including stage IVA and IVB). The median follow-up time for the current cohort was 65.2 months (range 11.4–77.2). Finally, there were 112 cancer-related deaths at the time of the last follow-up.

**Table 1:** Baseline characteristics of the patients according to the PDW and PLT

Variables	Total n =168	PDW (fL)		*P*	PLT (×10^9^/L)		*P*
		≤16.3 (n=87)	>16.3 (n=81)		≤266 (n=100)	>266(n=68)	
Age (years)				0.120			0.176
< 60	146	79	67		84	62	
≥ 60	22	8	14		16	6	
Gender				0.252			0.773
Male	119	65	54		70	49	
Female	49	22	27		30	19	
Smoking				0.615			0.485
Yes	59	29	30		33	26	
No	109	58	51		67	42	
BMI(kg/m2)				0.091			0.491
>23.7	82	37	45		51	31	
≤23.7	86	50	36		49	37	
Diabetes				0.290			0.053
Yes	11	4	7		3	8	
No	157	83	74		97	60	
Family history of NPC				0.735			0.245
Yes	18	10	8		13	5	
No	150	77	73		87	63	
KPS				0.176			0.069
≤80	76	35	41		51	25	
> 80	92	52	40		49	43	
Undifferentiated carcinoma				0.440			0.209
Yes	84	46	38		54	30	
No	84	41	43		46	38	
T stage				0.019			0.287
T1-2	80	49	31		51	29	
T3-4	88	38	50		49	39	
N stage				0.026			0.574
N0-1	60	38	22		34	26	
N2-3	108	49	59		66	42	
Clinical stage				< 0.001			0.574
I-II	28	24	4		18	10	
III-IVB	140	63	77		82	58	
CCRT				0.519			0.086
Yes	116	62	54		64	52	
No	52	25	27		36	16	
IMRT				0.533			0.147
Yes	126	67	59		79	47	
No	42	20	22		21	21	
EBV DNA				0.096			0.194
High	114	54	60		64	50	
low	54	33	21		36	18	

Abbreviations: PDW, platelet distribution width; PLT, platelet count; BMI, Body mass index; UC, Undifferentiated carcinoma; KPS, Karnofsky performance score; CCRT, concurrent chemoradiotherapy; IMRT, intensity-modulated radiotherapy; EBV: Epstein–Barr.

**Figure 1:**
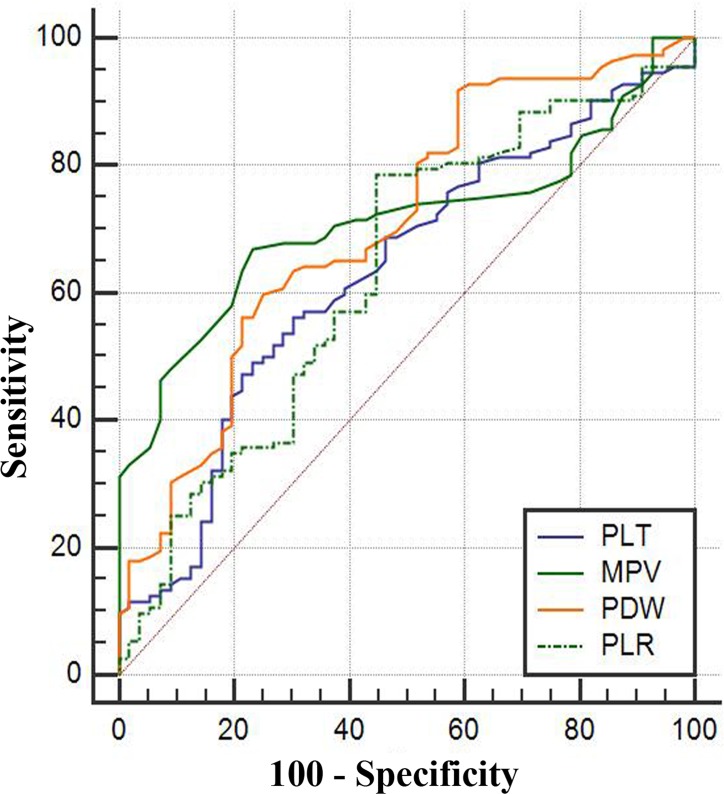
The ROC curves grouped by PLT, MPV, PDW and PLR ROC, receiver operating characteristic. Notes: The blue line represents PLT, the green line MPV, and the orange line PDW, and the imaginary line PLR.

According to the ROC curve analysis, the optimum cut-off values of the PLT, MPV, PDW, and PLR for the 5-year OS were 266 × 10^9^/L (sensitivity 49.1%, specificity 76.8%), 10.6 fL(sensitivity 67.0%, specificity 76.8%), 16.3 fL(sensitivity 59.8%, specificity 75.0%), and 130.22 (sensitivity 78.6%, specificity 55.4%), respectively. The areas under the curve (AUCs) for the PLT, MPV, PDW, and PLR were 0.632 (95% CI: 0.554–0.705, P = 0.003), 0.713 (95% CI: 0.638–0.780, P < 0.001), 0.707 (95% CI: 0.631–0.774, P < 0.001), and 0.632 (95% CI: 0.554–0.705, P = 0.004), respectively (Figure 1 and Table 2). Consistent with a previous report [5], we used 1500 copies/mL as the cut-off value, and all of the patients were divided into either low (< 1500 copies/mL) or high (≥ 1500 copies/mL) pretreatment EBV DNA groups.

**Table 2:** Receiver operating characteristic curve analyses showing the utility of platelet indices for NPC

Platelet indices	Cut-off values	AUC	Sensibility	Specificity	95% CI	P-value
PLT (×109/L)	266	0.632	49.1	76.8	0.554-0.705	0.003
MPV (fL)	10.6	0.713	67.0	76.8	0.638-0.780	<0.001
PDW (fL)	16.3	0.707	59.8	75.0	0.631-0.774	<0.001
PLR	130.22	0.632	78.6	55.4	0.554-0.705	0.004

Abbreviations: see Table 1.

The univariate Cox proportional hazards analyses revealed that the age at diagnosis (categorical variable), depth of invasion (T stage, T3–4 vs. T1–2), lymph node metastasis (N stage, N2–3 vs. N0–1), clinical stage (III–IV vs. I–II), platelet count (× 109/L) (> 266 vs. ≤ 266), MPV (fL) (> 10.6 vs. ≤ 10.6), PDW (fL) (> 16.3 vs. ≤ 16.3), PLR (> 130.22 vs. ≤ 130.22), and EBV DNA (high vs. low) were significantly associated with the OS (Table 3). However, the other parameters were not found to be correlated with the OS. All of the factors with P values of less than 0.05 in the univariate analyses were included in the subsequent multivariate Cox proportional hazards models.

**Table 3:** Result of the univariate analysis of overall survival in NPC patients

Characteristic	Hazard ratio	95% CI	P-value
Sex (male vs. female)	1.435	0.925-2.227	0.107
Age (≥60 vs. < 60 years)	1.719	1.035-2.852	0.036
Smoking (yes vs. no)	1.448	0.985-2.129	0.060
BMI (kg/m2) (>23.7 vs. ≤23.7)	1.335	0.917-1.943	0.132
Diabetes (yes vs. no)	1.767	0.856-3.647	0.124
Family history of NPC (yes vs. no)	0.986	0.563-1.728	0.962
KPS (≤80 vs. > 80)	1.168	0.805-1.696	0.414
Undifferentiated cancer (yes vs. no)	0.948	0.652-1.380	0.782
T stage(T3-4 vs. T1-2)	4.786	2.963-7.728	<0.001
N stage (N2–3 vs. N0–1)	4.099	2.536-6.651	<0.001
Clinical stage (III–IVB) vs. (I–II)	43.441	10.284 – 183.507	<0.001
CCRT (no vs. yes)	0.936	0.620-1.413	0.753
IMRT (no vs. yes)	1.292	0.839-1.988	0.245
PLT (>266 vs. ≤266) (×109/L)	1.979	1.361-2.877	<0.001
MPV (>10.6vs.≤10.6) fL	0.385	0.257-0.577	<0.001
PDW (>16.3 vs. ≤16.3) fL	2.986	2.030 – 4.394	<0.001
PLR (>130.22 vs. ≤130.22)	2.890	1.835 – 4.553	<0.001
EBV DNA (high vs. low) (copies/mL)	2.192	1.418 – 3.388	<0.001

Abbreviations: see Table 1.

In the multivariate analyses, we demonstrated that the PDW (HR 2.362, 95% CI: 1.554–3.590, P < 0.001), PLT (HR 2.017, 95% CI: 1.329–3.060, P = 0.001), T stage (HR 2.768, 95% CI: 1.603–4.779, P < 0.001), N stage (HR 2.165, 95% CI: 1.245–3.765, P = 0.006), clinical stage (HR 9.534, 95% CI: 1.998–45.498, P = 0.005), and EBV DNA (HR 1.619, 95% CI: 1.024–2.560, P = 0.039) were independent prognostic factors for NPC patients (Table 4).

**Table 4:** Multivariate Cox proportional hazards regression analysis

Characteristic	Hazard ratio	95% CI	P-value
Age (≥60 vs. < 60 years)	1.696	0.927-3.104	0.087
T stage(T3-4 vs. T1-2)	2.768	1.603-4.779	< 0.001
N stage (N2–3 vs. N0–1)	2.165	1.245-3.765	0.006
Clinical stage (III–IVB) vs. (I–II)	9.534	1.998-45.498	0.005
PLT (>266 vs. ≤266) (×109/L)	2.017	1.329-3.060	0.001
MPV (>10.6vs.≤10.6) fL	0.645	0.404-1.028	0.065
PDW (>16.3 vs. ≤16.3) fL	2.362	1.554-3.590	< 0.001
PLR (>130.22 vs. ≤130.22)	1.523	0.933-2.487	0.092
EBV DNA (high vs. low) (copies/mL)	1.619	1.024-2.560	0.039

Abbreviations: see Table 1.

The relationship between the PDW, PLT, and clinicopathological characteristics in NPC patients is shown in Table 1. There were no differences in the age distribution (P = 0.120), gender (P = 0.252), smoking history (P = 0.615), body mass index (P = 0.091), diabetes incidence (P = 0.290), family history of NPC (P = 0.735), KPS (P = 0.176), undifferentiated carcinoma incidence (P = 0.440), CCRT (P = 0.519), IMRT (P = 0.533), and EBV DNA (P = 0.096) with regard to the PDW. However, the PDW was associated with the T stage (P = 0.019), N stage (P = 0.026), and clinical stage (P < 0.001). Moreover, none of the clinicopathological features were significantly associated with the PLT.

**Figure 2:**
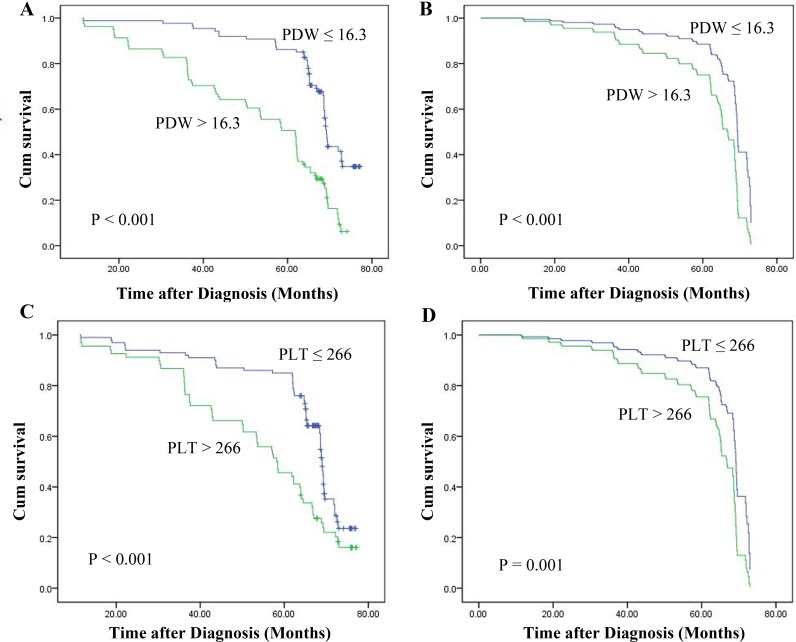
Kaplan-Meier survival curves for NPC patients according to whether they show high or lower **(A)** PDW and **(C)** PLT levels. Multivariate Cox survival curves for NPC patients according to whether they show high or lower **(B)** PDW and **(D)** PLT levels.

The 5-year OS rate in this cohort was 69.05%, while the 5-year overall survival rates in those patients with PDW ≤ 16.3 fL and PDW > 16.3 fL were 86.21% and 50.62%, respectively. The results of the Kaplan-Meier and log-rank tests showed that the OS of the NPC patients with PDWs greater than 16.3 fL was shorter than that of the patients with PDWs less than or equal to 16.3 fL (P < 0.001). In addition, the OS of those patients with an increased PLT was also significantly shorter than in those with lower PLT levels (P < 0.001). Additionally, an adjusted Cox proportional hazard survival curve was used to investigate the effects of the PDW and PLT on survival. The outcomes were the same as those obtained while using the Kaplan-Meier method (Figure 2).

## DISCUSSION

To the best of our knowledge, this is the first study to investigate the prognostic value of the pretreatment PDW in NPC patients, and the first to systematically evaluate the relationships between the PLT, MPV, PDW, PLR, and the prognosis of NPC. This study revealed that a PDW with a cut-off 16.3 fL and a PLT with a cut-off 266 × 10^9^/L had independent prognostic significances for the OS in NPC patients.

Platelets, a blood component, have been generally recognized as mediating tumor cell growth, metastasis, and angiogenesis. Activated platelets are capable of interacting with cancer cells through paracrine signaling or direct contact, consequently promoting tumor cell growth and survival [19]. An elevated blood platelet count is a common phenomenon in many kinds of malignancies and is linked to reduced survival in patients with various tumor types, including lung, breast, gastric, colorectal, pancreatic, brain, endometrial, and ovarian cancer. Although these results somewhat overlapped with those of the present study, our present study confirmed the key role of activated platelets in NPC using a simple platelet parameter. These data are also in accordance with the present knowledge that antiplatelet therapy is considered to be one portion of adjuvant cancer therapy [8].

It is now generally recognized that the PDW can not only measure platelet volume heterogeneity, but also reflect platelet activity. Recently, one study reported that a reduced PDW is an unfavorable predictive factor for non-small cell lung cancer patient survival [13]. Contrarily, an elevated PDW has been demonstrated to have a poor prognostic impact in melanoma [20], thyroid cancer [21], colorectal cancer [12], and laryngeal cancer [11]. However, the clinical value of the PDW has not yet been reported in NPC patients. Consistent with most studies, our results showed that an increased PDW was not only associated with the T stage, N stage, and clinical stage, but also significantly associated with a poorer OS in NPC patients. Moreover, an elevated PDW was an independent prognostic index for the OS in NPC patients.

New study has demonstrated that a high pretreatment MPV level in invasive breast cancer patients is a potential predictive factor and significant independent prognostic factor [14]. The study conducted by Omar et al. [22] reported that an increased MPV level may be used as a prognostic biomarker to estimate a poor OS in patients with lung cancer. However, the researchers found that the MPV levels were significantly increased in those patients with gastric ulcers and in the control subjects when compared with the gastric cancer patients [23]. This result seems to suggest that the MPV is a protective factor in gastric cancer. Moreover, this retrospective cohort study is the first study to investigate the prognostic value of pretreatment MPV levels in NPC patients. The results revealed that those patients with high MPV levels had 0.645 times the risk of death when compared to those with a low MPV levels, albeit the difference was not statistically significant after the multivariate analysis.

A previous study showed that elevated PLR values were associated with poor cancer-specific survival, OS, and distant metastasis free survival in patients with NPC [18]. In this study, the results of the multivariate analysis revealed that those patients with high PLR levels had 1.52 times the risk of death when compared to those with low PLR levels; however, the difference was not statistically significant (P > 0.092). The discrepancies above may be attributed to the different tumor tissues, different sample sizes, and variability in the measurement methods. Of course, there is the fact that we have additionally adjusted for some potential prognostic factors, including the PLT and PDW.

At present, the biological mechanisms underlying the correlation between the PDW, PLT, and tumorigenesis or progression in NPC remains poorly understood. The platelet volume is determined during both megakaryopoiesis and thrombopoiesis. In megakaryocytic maturation, the platelet production and platelet size can be modulated by cytokines, such as interleukin-6 (IL-6), the granulocyte colony stimulating factor, and the macrophage colony stimulating factor [24]. In addition, the tumor-associated production of the granulocyte-macrophage colony stimulating factor or thrombopoietin mediated by IL-6 is considered to be responsible for the increase in the PLT observed in cancer patients [25]. There is consistent evidence that IL-6 is overexpressed in EBV-infected nasopharyngeal epithelial cancer cells [26]. By activating the IL-6-associated transcription factors (nuclear factor kappa-light-chain-enhancer of activated B cells and signal transducer and activator of transcription 3), the enhanced circulating IL-6 not only contributes to NPC's proliferative properties but also mediates platelet generation. Furthermore, elevated platelet levels promote cancer progression and metastasis by protecting the circulating tumor cells from immune surveillance and death [27]. Another reasonable mechanism is that platelets facilitate a hypercoagulable state in cancer. The increased number of platelets produces a procoagulant microenvironment that enables the cancer cells to cover themselves with platelets and escape the host's immune system [28].

There were some study limitations that should be addressed. First, our study may have been restricted by its retrospective design. Second, the number of subjects included in our study was relatively small, and the power of the statistical analysis was relatively weak; therefore, larger studies are warranted to validate our findings. Finally, all of the included studies were from Chinese populations; consequently, the conclusion might be limited to East Asian individuals.

In summary, the current study results suggest that the PDW and PLT could be new independent prognostic factors in NPC patients. However, additional investigations are warranted to fully understand the potential mechanism. These easily measured and inexpensive biomarkers may be used as practical predictors in daily clinical practice in the future.

## MATERIALS AND METHODS

### Patient recruitment and data collection

A total of 168 NPC patients were admitted to the Department of Oncology of the Affiliated Hospital of XiangNan University between January 2011 and June 2012. All of the included patients met the following criteria: (1) histological confirmation of NPC and no previous anticancer treatment; (2) no distant metastasis; (3) had received complete blood differential count records within 3 days prior to treatment; (4) available clinical information. The exclusion criteria were as follows: (1) Karnofsky Performance Scale (KPS) score < 70; (2) had other previously treated and/or synchronous malignancies; (3) the presence of a concomitant disease that can affect the platelet count, including severe hypertension, autoimmune disease, inflammation, liver cirrhosis, splenic disease, and a history of blood transfusion.

The pretreatment hematological parameters were measured via a Beckman Coulter LH750 automated analyzer (Beckman Coulter, California, USA). The EBV DNA concentrations were obtained using a quantitative polymerase chain reaction (DA 7600; Da-an, Zhongshan, China) before treatment, as previously reported [29].

This study was approved by the Institutional Review Board of the Affiliated Hospital of XiangNan University.

### Staging, treatment, and follow-up

All of the patients were reassigned based on the 7th Edition of the American Joint Committee on Cancer (AJCC) cancer staging system for NPC as assessed via the compilation of the magnetic resonance imaging, chest radiograph, abdominal ultrasonography, whole-body bone scanning, and/or positron emission tomography-computed tomography results.

The treatment strategies for all of the patients were based on the National Comprehensive Cancer Network Guidelines. All of the patients received definitive radiotherapy (RT) using three-dimensional conformal RT (3D-CRT) and intensity-modulated RT (IMRT) techniques. Those patients with stage I–II malignancies were treated by RT alone or with concurrent chemoradiotherapy (CCRT). Those patients with stage III–IVB malignancies were treated with CCRT with or without induction/adjuvant chemotherapy. The CCRT regimen was the use of 30 mg/m^2^ of cisplatin every week for 6–7 cycles during the RT period. The regimen for the induction/adjuvant chemotherapy included cisplatin (75 mg/m^2^ on day 1) and 5-fluorouracil (1,000 mg/m^2^ via a 96-hour continuous infusion from days 2–5) administrated every 21 days for 2-3 cycles.

After completing treatment, the patients were followed up every three months for the first three years, with the intervals gradually increasing to 6–12 months after three years. The last follow-up date was June 30, 2017. The overall survival (OS) was calculated from the date of the diagnosis to the date of death or the last follow-up. The median follow-up time was 65.2 months.

### Statistical analysis

The statistical analysis was performed using the Statistical Program for Social Sciences (SPSS) software, version 16.0 (SPSS Inc., Chicago, IL, USA). The optimal cut-off values of the platelet indices were determined by using a receiver operating characteristic (ROC) curve. The univariate and multivariate survival analyses were determined using the Cox proportional hazards model. The correlations between the potential prognostic predictors and the clinicopathological variables were evaluated via the chi-squared test. The Kaplan-Meier method and the corresponding log rank test were used for the survival analyses of the categorical variables. All of the tests were two-tailed, and the level of significance was P < 0.05.
